# Nuclear trapping of inactive FOXO1 by the Nrf2 activator diethyl maleate

**DOI:** 10.1016/j.redox.2018.09.010

**Published:** 2018-09-14

**Authors:** Andrea Gille, Abdullah Turkistani, Dimitrios Tsitsipatis, Xiaoqing Hou, Sarah Tauber, Ingrit Hamann, Nadine Urban, Katrin Erler, Holger Steinbrenner, Lars-Oliver Klotz

**Affiliations:** a Institute of Nutritional Sciences, Nutrigenomics, Friedrich-Schiller-Universität Jena, D-07743 Jena, Germany; b Faculty of Pharmacy and Pharmaceutical Sciences, University of Alberta, Edmonton, AB, Canada

**Keywords:** FOXO, DAF-16, Glutathione, Stress response, Nrf-2, Thiols, Nuclear export

## Abstract

Diethyl maleate (DEM), a thiol-reactive α,β-unsaturated carbonyl compound, depletes glutathione (GSH) in exposed cells and was previously shown by us to elicit a stress response in *Caenorhabditis elegans* that, at lower concentrations, results in enhanced stress resistance and longer lifespan. This hormetic response was mediated through both the Nrf2 ortholog, SKN-1, and the forkhead box O (FOXO) family transcription factor DAF-16. As FOXO signaling is evolutionarily conserved, we analyzed here the effects of DEM exposure on FOXO in cultured human cells (HepG2, HEK293). DEM elicited nuclear accumulation of GFP-coupled wild-type human FOXO1, as well as of a cysteine-deficient FOXO1 mutant. Despite the nuclear accumulation of FOXO1, neither FOXO1 DNA binding nor FOXO target gene expression were stimulated, suggesting that DEM causes nuclear accumulation but not activation of FOXO1. FOXO1 nuclear exclusion elicited by insulin or xenobiotics such as arsenite or copper ions was attenuated by DEM, suggesting that DEM interfered with nuclear export. In addition, insulin-induced FOXO1 phosphorylation at Thr-24, which is associated with FOXO1 nuclear exclusion, was attenuated upon exposure to DEM. Different from FOXO-dependent expression of genes, Nrf2 target gene mRNAs were elevated upon exposure to DEM. These data suggest that, different from *C. elegans*, DEM elicits opposing effects on the two stress-responsive transcription factors, Nrf2 and FOXO1, in cultured human cells.

## Highlights

• Diethyl maleate (DEM) causes FOXO1 nuclear accumulation in HepG2 or HEK293 cells.
• DEM also elicits nuclear accumulation of the FOXO-ortholog, DAF-16, in *C. elegans*.
• Nevertheless, DEM does not elicit expression of FOXO, but of Nrf2 target genes.
• Insulin-induced FOXO1 phosphorylation and nuclear exclusion are attenuated by DEM.
• Nuclear accumulation of FOXO1 is due to interference with nuclear export machinery.

## Graphical abstract

**Figure:**
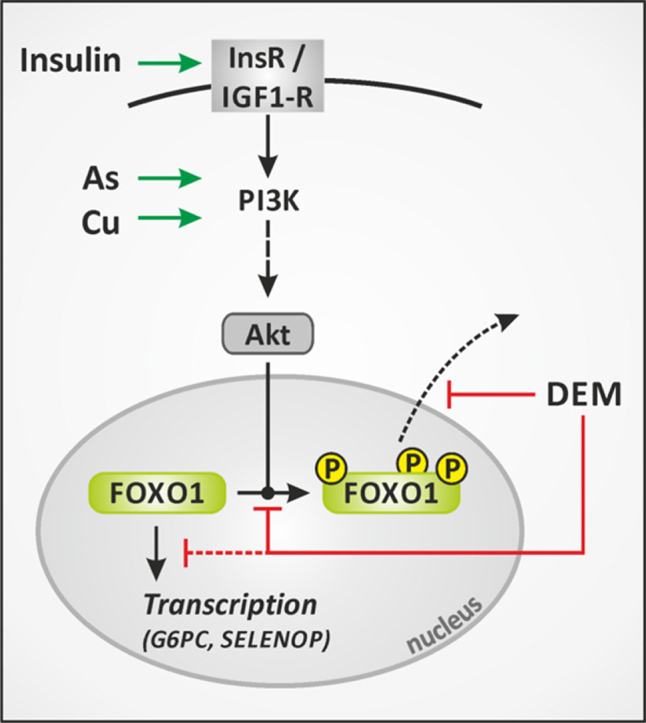

## 1 Introduction

The intracellular tripeptide thiol, glutathione (γGlu-Cys-Gly; GSH), is generally known as a crucial contributor to cellular antioxidant defense systems as well as to xenobiotic metabolism [1]. It was therefore previously tested for its role in the regulation of stress resistance and life span in the model organism, *Caenorhabditis elegans*. In line with its role in antioxidant defense, a decreased stress resistance of *C. elegans* was originally expected to result from GSH depletion. Unexpectedly, however, a non-linear relationship between GSH levels and stress resistance was observed, indicating that a moderate decrease of GSH in *C. elegans* may in fact enhance, rather than diminish, resistance against oxidative stress [2].

Diethyl maleate (DEM), an α,β-unsaturated carbonyl compound that is frequently used for the depletion of cellular GSH, and a known stimulator of Nrf2 signaling in mammalian cells [3], [4], [5], also elicited an increase in stress resistance in *C. elegans* [2]. Not only SKN-1, the ortholog of Nrf2, but also DAF-16, the *C. elegans* ortholog of mammalian FOXO transcription factors, was demonstrated to contribute to this effect [2].

FOXO transcription factors are evolutionarily conserved major regulators of cellular metabolic processes, including fuel metabolism, antioxidant defense and cell death [6], [7]. Insulin, through phosphoinositide 3′-kinase (PI3K)-dependent stimulation of FOXO phosphorylation by the Ser/Thr-kinase Akt, causes inactivation and nuclear exclusion of three of the four mammalian FOXO isoforms (FOXOs 1, 3 and 4) [8]. As FOXOs were previously demonstrated in mammalian cells to be regulated not only by insulin, but also by stressful stimuli, such as by hydrogen peroxide [9] or the thiophilic agents Cu^2+^ and arsenite [10], [11], we asked whether exposure to the thiol depleting agent DEM would result in a similar modulation of FOXO signaling in mammalian cells, and whether this occurs with consequences for FOXO target gene expression that are similar to the observations in *C. elegans*.

We here demonstrate that indeed DEM affects FOXO1 subcellular localization in cultured human cells, but that the consequences of DEM exposure differ from those observed in *C. elegans* as FOXO-dependent gene expression was not elicited by a mere depletion of GSH. Rather, we propose that DEM interferes with nucleocytoplasmic shuttling of FOXOs.

## 2 Materials and methods

### 2.1 Materials

SP600125 was purchased from Selleck Chemicals (Houston, TX, USA); all other chemicals were purchased from Sigma-Aldrich (Munich, Germany) and Carl Roth (Karlsruhe, Germany) unless stated otherwise. Primers were obtained from Life Technologies (Darmstadt, Germany).

### 2.2 Cell culture, transfection, plasmids

HepG2 human hepatoma cells and HEK293 human embryonic kidney cells were obtained from the German Collection of Microorganisms and Cell Cultures (DSMZ, Braunschweig, Germany). HEK293 cells were held in Dulbecco's modified Eagle's medium (DMEM, low glucose; Sigma, Munich, Germany, Cat# D6046), supplemented with 10% (v/v) fetal bovine serum (Biochrom, Berlin, Germany), 100 U/mL penicillin, 100 μg/mL streptomycin (Sigma); HepG2 cells were held likewise, with non-essential amino acids (in MEM; Sigma, Cat# M7145) added to the medium. Cells were maintained at 37 °C in a humidified atmosphere with 5% (v/v) CO_2_. For DNA binding assays (Fig. 3B) DMEM with 4500 mg/l glucose was used (Sigma, Cat# D6429), and for experiments in Figs. 4B-D, 5A-B and 6A, HepG2 cells were held in DMEM with 4500 mg/l glucose and 2 mM glutamine (Sigma-Aldrich) supplemented with 10% (v/v) FCS (PAA, Etobicoke, ON, Canada), penicillin/streptomycin and non-essential amino acids.

Transfection of HepG2 cells was performed using GenJet (SignaGen Laboratories, Rockville, MD, USA) or nanofectin (PAA, Etobicoke, ON, Canada) according to the manufacturers’ instructions. For transfection of HEK293 cells TurboFect reagent (Thermo Scientific, Waltham, MA, USA) was used according to the manufacturer's recommendations.

Plasmids containing cDNA encoding human wildtype FOXO1 [FOXO1(WT)] and human FOXO1 with all seven cysteines mutated into serines [FOXO1(C#1-7S)] coupled to GFP were generated as described previously [12]. Briefly, wildtype and mutated FOXO1 sequences were cloned into two different GFP vectors, pcDNA-DEST53 (Invitrogen) or pEGFP-C1 (Clontech), yielding pcDNA-GFP-hFOXO1(WT) and pEGFPC1-hFOXO1(WT), respectively, as well as the mutant versions, pcDNA-GFP-hFOXO1(C#1-7S) and pEGFPC1-hFOXO1(C#1-7S).

### 2.3 Analysis of subcellular FOXO1 localization

HepG2 or HEK293 cells transfected with plasmids coding for human FOXO1 variants N-terminally coupled to GFP were analyzed by fluorescence microscopy following transfection (usually approx. 24 h post-transfection). Visibly GFP-positive cells were grouped into three categories with respect to the predominant subcellular localization of the GFP signal (“cytoplasmic”, “cytoplasmic/nuclear” or “nuclear”). Time-course analyses of subcellular relocalization (Fig. 5C) were performed as follows: HepG2 cells were grown on 6-channel-microscopy slides with a coverslip bottom (“µ-Slides VI 0.4”; ibidi, Martinsried, Germany, Cat# 80606) covered with collagen. 16–22 h following transfection of cells with GFP plasmids (GenJet reagent, added already to the cell suspension prior to their application to slides), cells were exposed to insulin and/or DEM (or the respective solvent controls). The microscopy slides were placed in an incubator box (okolab, Ottaviano, Italy) and held at 37 °C/5% CO_2_ while fluorescence microscopic images were taken at multiple time points (Nikon Eclipse Ti fluorescence microscope).

### 2.4 C. elegans maintenance and treatment

The TJ356 zIs356 [daf-16p::daf-16a/b::GFP + rol-6] *C. elegans* strain was provided by the *Caenorhabditis* Genetics Center (CGC, University of Minnesota, USA), which is supported by the National Institutes of Health-Office of Research Infrastructure Programs. *E. coli* strain OP50 was also received from CGC. Nematodes were grown, maintained and treated at 20 °C on nematode growth medium (NGM) agar plates spotted with *E. coli* OP50 as food source, as previously described [2]. Stock solutions of diethyl maleate (DEM) were prepared in DMSO. DEM or the solvent control (0.1% DMSO) were added directly to the agar during preparation of plates. 24 h after synchronization, nematodes of the transgenic strain TJ356 stably expressing a DAF-16::GFP fusion protein were transferred to NGM agar plates containing the respective compound or solvent control for an additional 24 h. Subsequently, around 40 L3 larvae of each group were placed on microscope slides coated with 3% agarose, anaesthetized with 10 mM sodium azide, and covered with coverslips. Cellular localization of DAF-16 was analyzed by fluorescence microscopy on an Axio Observer D1 fluorescence microscope (Zeiss, Göttingen, Germany) using appropriate filters (ex. 472 ± 30 nm, em. 520 ± 35 nm).

### 2.5 Western blotting

Western blotting was performed according to standard procedures [11], using the following primary antibodies: phospho-Jun (Ser63), phospho-SAPK/JNK (Thr183/Tyr185) Rabbit mAb (98F2), phospho-FoxO1 (Thr24)/FoxO3a (Thr32), FoxO1 (C29H4) Rabbit mAb (all from Cell Signaling Technology, Danvers, MA, USA), GAPDH (Millipore, Billerica, MA, USA). Incubation with secondary antibody [horseradish peroxidase (HRP)-conjugated anti-rabbit IgG or HRP-coupled anti-mouse IgG (GE-Healthcare, Piscataway, USA)] was followed by detection using chemiluminescent HRP substrate. Images were acquired using an ImageQuant LAS 4000 mini system (GE Healthcare Bio-Sciences).

### 2.6 Determination of glutathione levels

GSH was determined by HPLC (Jasco, Gross-Umstadt, Germany) after derivatization of thiols with orthophtaldialdehyde (OPA) and fluorometric detection as described [2]. Cells grown to approx. 75% confluence in cell culture dishes (growth area: 58 cm^2^) were scraped off the dishes in 1 mL of ice-cold 0.01 N HCl. Suspensions were frozen at − 80 °C for approx. 20 h, thawed, resuspended 10 times, then centrifuged to separate debris (4 °C, 18,000×*g*). After centrifugation, aliquots of 50 µl were prepared from the supernatant and stored at − 80 °C until further use. Four independent experiments were performed, and two different aliquots of the same supernatant were determined in each of the experiments for each data point. Protein content was determined from another of the aliquots in a bicinchoninic acid (BCA) assay (Thermo Fisher Scientific). Proteins were precipitated by addition of 25 µl of cold 2 N perchloric acid to 50 µl of the supernatant, followed by incubation on ice for 1 min. This mixture was neutralized by addition of 200 µl of 0.5 M sodium phosphate buffer (pH 7.0), followed by centrifugation for 10 min at 4 °C. 50 µl of the neutralized supernatant was used for derivatization with 50 µl of OPA [0.15 M in 0.1 M sodium borate, pH 9]. Separation was performed by gradient elution on a ZORBAX Bonus RP column (4.6 × 250 mm; Agilent) at a flow rate of 1 mL/min. Eluents were (A) 98% of 50 mM sodium acetate (pH 7)/2% acetonitrile (VWR, Fontenay-sous-Bois, France) and (B) 80% acetonitrile/20% 50 mM sodium acetate (pH 7.0). Peaks were detected at 420 nm after excitation at 340 nm. GSH was normalized to protein content of the respective sample. Glutathione disulfide levels were measured analogously, after chemical masking of GSH using N-ethyl maleimide (NEM), followed by blocking residual NEM and reduction of GSSG to GSH, which was then detected as above.

### 2.7 Quantitative (real-time) RT-PCR

For quantitative reverse transcription PCR (qRT-PCR) total RNA from HepG2 cells was isolated using an RNeasy Mini kit (Qiagen, Hilden, Germany) according to the manufacturer's instructions. 1 µg of total RNA was reverse-transcribed using RevertAid reverse transcriptase (Thermo Scientific) according to the manufacturer's instructions, and subjected to qPCR analysis on a CFX Connect cycler (Bio-Rad Laboratories AG, Munich, Germany) using SsoAdvanced Universal SYBR Green Supermix (Bio-Rad). The housekeeping gene HPRT-1 transcript was used for normalization of mRNA levels. The employed primers for PCR are listed in Table 1.

**Table 1:** Primers used for Real-Time RT-PCR analysis.

**Gene**	**Gene ID**	**Primers (5′→3′)**
**G6PC**	NM_000151	TTCCCTGTAACCTGTGAGACTG
		AGATGGAAAGAGTAGATGTGACCAT
**SELENOP**	NM_005410.3	GGAGCTGCCAGAGTAAAGCA
		ACATTGCTGGGGTTGTCAC
**HPRT**	NM_000194.2	GGGGACATAAAAGTAATTGGTGGAG
		CTGACCAAGGAAAGCAAAGTCTG
**G6PD**	NM_001042351.2	GCAAACAGAGTGAGCCCTTC
		GGCCAGCCACATAGGAGTT
**HMOX1**	NM_002133.2	AGACTGCGTTCCTGCTCAAC
		GGCTCTGGTCCTTGGTGTC
**NQO1**	NM_000903.2	GCTCACCGAGAGCCTAGTTC
		TCCTCTCTGAGTGAGCCAGT

### 2.8 FOXO1 DNA binding

DNA binding activity was analyzed employing an ELISA-based FOXO-DNA binding assay (TransAM FKHR, Active Motif, La Hulpe, Belgium) according to the manufacturer's instructions. In brief, cells were harvested and nuclear protein extracted using a nuclear extract kit (Active Motif) according to the manufacturer's protocol. Nuclear extracts were applied to 96-well plates coated with oligonucleotides containing FOXO-DNA binding elements. Bound (i.e., active) FOXO was then detected using an antibody directed against FOXO1, the binding of which was assessed employing a secondary antibody conjugated with HRP.

## 3 Results

### 3.1 Nuclear accumulation of FOXO1-GFP in cells exposed to DEM

**Fig. 1:**
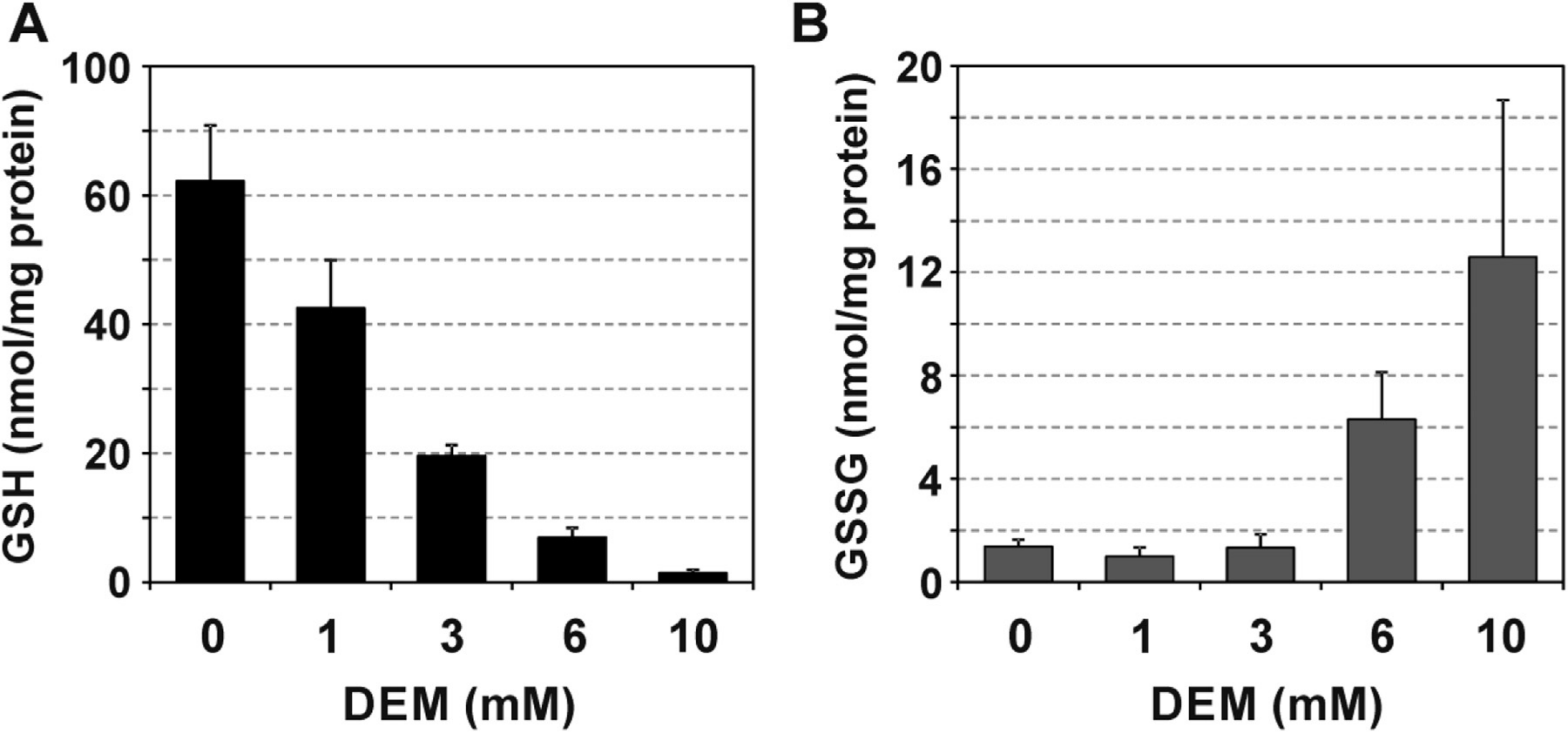
Glutathione depletion and oxidation in HepG2 cells exposed to diethyl maleate. Analysis of glutathione (GSH, A) and glutathione disulfide (GSSG, B) content of hepatoma cell lysates following exposure to the given concentrations of DEM or solvent (DMSO, “0 mM DEM”) for 120 min. Data are means of four independent experiments + SD.

Exposure of cultured HepG2 human hepatoma cells to DEM for up to 2 h resulted in a dose-dependent general thiol depletion (data not shown), as well as a depletion of glutathione (Fig. 1**A**). At higher concentrations of DEM, glutathione depletion coincided with an increased cellular content of glutathione disulfide (Fig. 1**B**), indicating that an intracellular oxidative environment was established under these conditions.

**Fig. 2:**
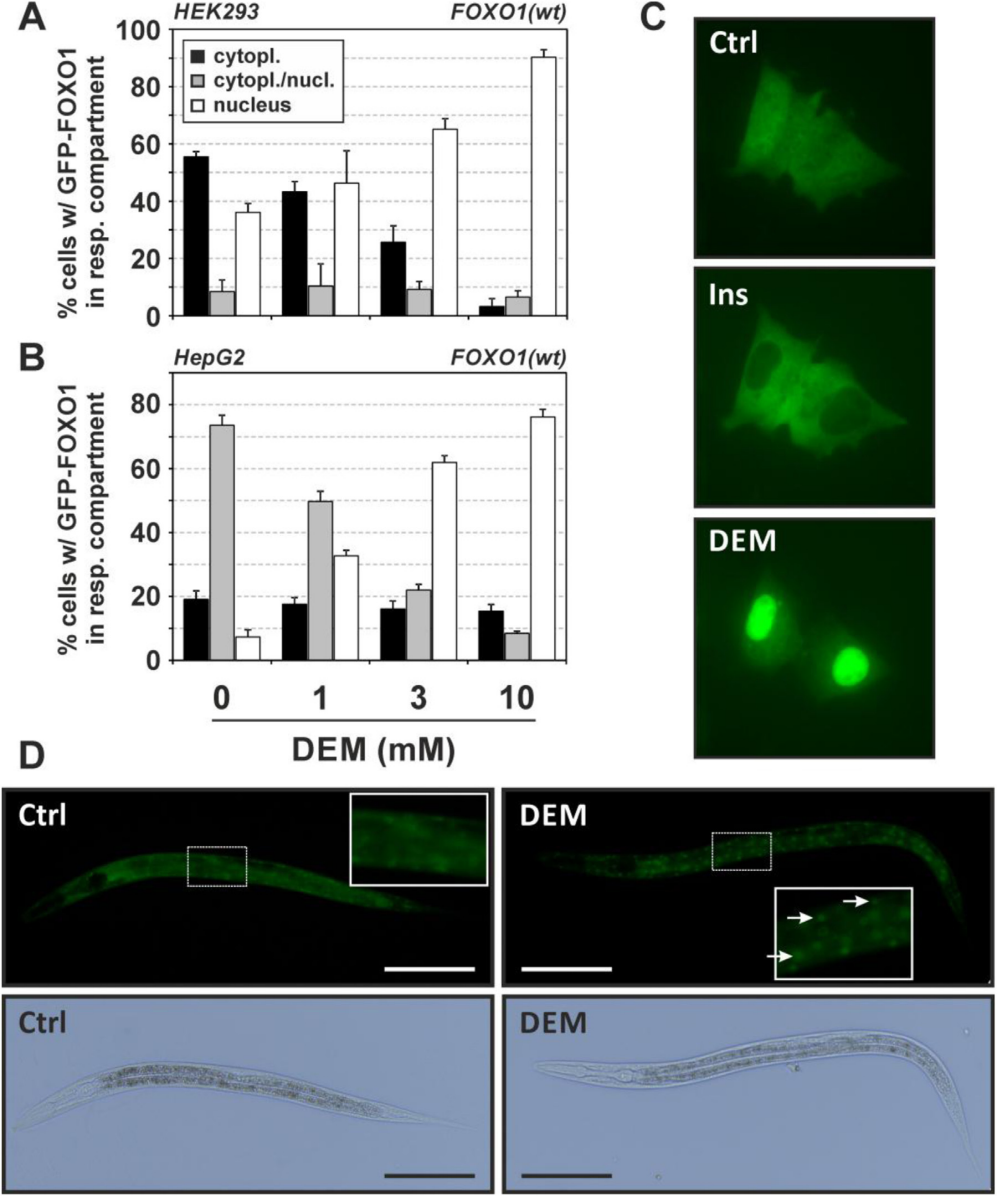
Diethyl maleate causes nuclear accumulation of FOXO1. **(A, B)** HEK293 or HepG2 cells transiently expressing GFP-coupled human FOXO1 (wildtype) were exposed to the given concentrations of DEM (“0”: solvent control, DMSO) for 30 min, followed by analysis of subcellular distribution of GFP-FOXO1. At least 200 cells were categorized for each independent experiment with respect to the predominant subcellular localization of GFP-FOXO1. Data are presented as means of three independent experiments + SD. **(C)** Images of HepG2 cells expressing GFP-FOXO1 prior to (Ctrl.) and after addition of insulin (Ins, 100 nM) and DEM (3 mM) as described in Fig. 5. The images shown provide examples of cells with GFP-FOXO1 in both nuclear and cytoplasmic compartments (Ctrl), predominantly cytoplasmic (insulin) and predominantly nuclear (DEM) localization. **(D)** Age-synchronized L1 larvae of the *C. elegans* TJ356 strain stably expressing a DAF-16::GFP fusion protein were transferred to NGM agar plates supplemented with DEM at 1 mM. 0.1% DMSO was used as control (Ctrl); exposure was for 24 h. Examples of worms with predominantly cytoplasmic (left) and nuclear localization (right, see arrows, inset) of DAF-16::GFP are shown. The experiment was performed at least three independent times. Bar = 100 µm.

We analyzed FOXO1 subcellular localization upon exposure to DEM in two different types of cultured human cells, HepG2 cells and HEK293 human embryonic kidney cells, using GFP-FOXO1 constructs. We used these two cell types because of their different patterns in basal subcellular FOXO1 distribution that correlates with basal Akt activity and FOXO1 phosphorylation levels as previously described [12]: Whereas HEK293 cells (with a higher basal Akt activity status) have a higher fraction of cells with predominantly cytoplasmic FOXO1 under basal growth conditions than HepG2 cells (black bars in Fig. 2**A-B**), the latter have a higher percentage of cells in an intermediate state with respect to FOXO1 distribution (gray bars), as seen also in Fig. 2**C** (top picture). Following DEM exposure, we detected rapid and dose-dependent nuclear accumulation of GFP-FOXO1 in both cell types (Fig. 2**A-C**). In contrast to DEM, insulin elicited the expected FOXO nuclear exclusion (Fig. 2**C**).

In line with our previous findings that DEM may elicit stress resistance in *C. elegans*, and that DAF-16, the *C. elegans* FOXO ortholog, is required for this effect [2], exposure to DEM of *C. elegans* expressing a GFP-tagged version of DAF-16, also elicited nuclear accumulation (visible as green dots) of the transcription factor (Fig. 2**D**).

**Fig. 3:**
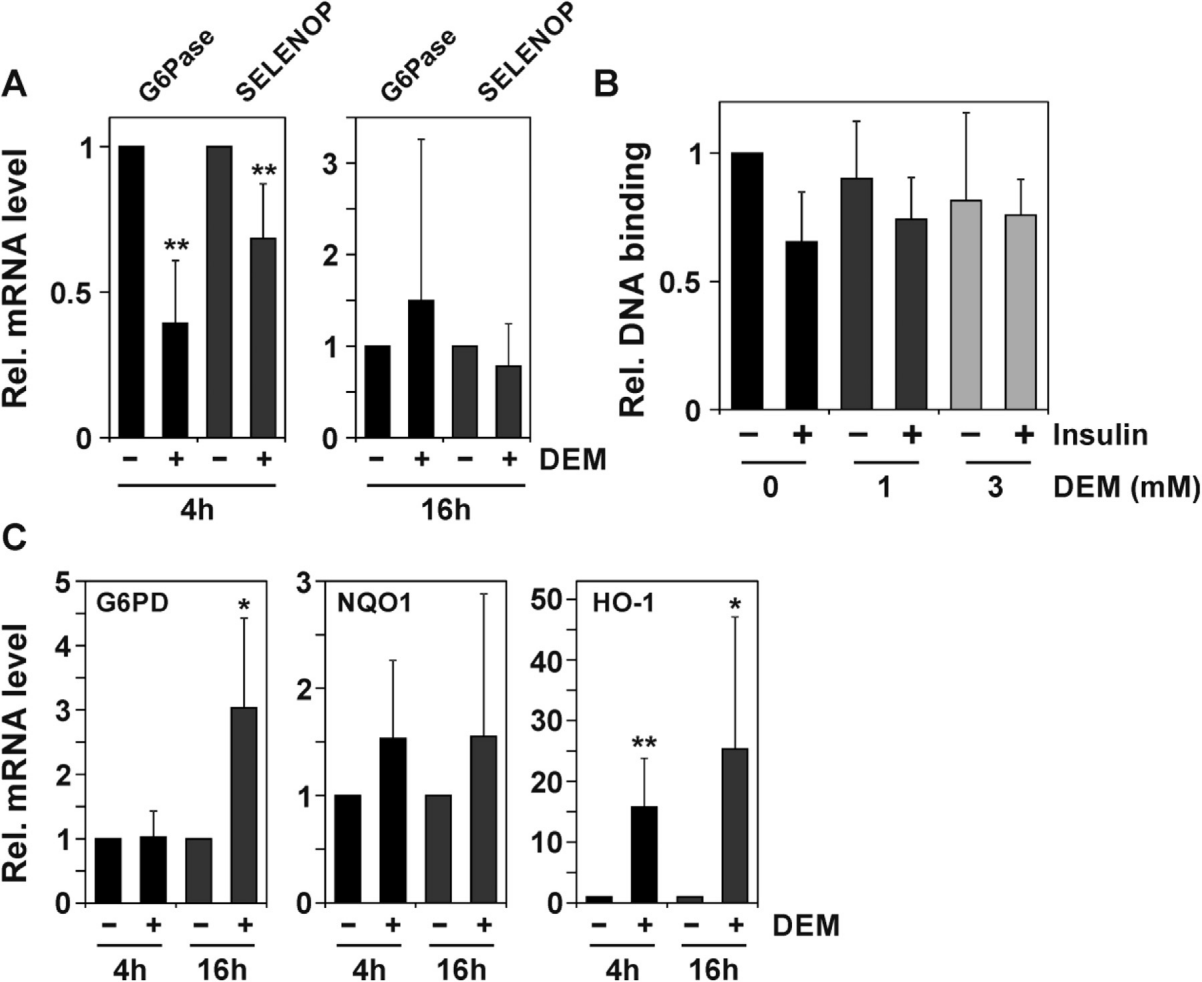
Diethyl maleate stimulates Nrf2 but not FOXO-dependent gene expression. HepG2 cells were exposed to DEM (1 mM) for 4 h or 16 h in serum-free DMEM **(A)**, followed by analysis of G6PC and SELENOP mRNA levels by qRT-PCR. Data are means of five independent experiments + SD. **(B)** HepG2 cells, grown to approx. 75% confluence, were held on serum-free medium for 18 h, followed by exposure to DEM at the given concentrations for 2 h. If indicated, this was followed by a 30 min incubation with insulin (100 nM). Nuclear extracts were prepared and binding of endogenous FOXO1 to an oligonucleotide containing FOXO binding elements analyzed in an ELISA-based approach. Data are means of three independent experiments + SD. **(C)** HepG2 cells were exposed to DEM for 4 h or 16 h, followed by analysis of Nrf2 target gene mRNA levels by qRT-PCR. Data are means of five independent experiments + SD. Statistical significance was assessed using Student's *t*-test: *P < 0.05, **P < 0.01 vs. respective control.

In order to test whether nuclear accumulation of FOXO1 as elicited by exposure to DEM also resulted in stimulation of FOXO target gene expression in HepG2 cells, we tested for modulation of mRNA levels of FOXO targets in cells exposed to DEM. We chose to test for glucose 6-phosphatase (G6Pase; *G6PC*) as well as selenoprotein P (*SELENOP*) mRNA levels in cells exposed to DEM. These two FOXO-regulated genes serve as indicators of FOXOs regulating gluconeogenesis and antioxidant defense, respectively [13], [14], [15]. As shown in Fig. 3**A**, neither of these mRNAs is upregulated upon exposure of cells to DEM. In fact, short-term exposure elicited a strong downregulation of G6Pase mRNA and resulted in lower SELENOP mRNA levels.

Despite the transiently lower mRNA levels of FOXO1 target genes upon DEM exposure, the capability of FOXO1 to bind DNA was not significantly altered: only a trend towards less DNA binding capability of FOXO1 was observed in cells exposed to DEM (Fig. 3**B**). This suggests that, while this slight attenuation of DNA binding may contribute to the lack of induced FOXO target gene expression, the major contributor in this respect is at a later stage, e.g. an attenuation of the interaction with transcriptional coregulators.

Moreover, the effect of DEM is not a general inhibitory effect on transcription, as exposure to DEM elicited an induced expression of Nrf2 target genes, including *G6PD*, *NQO1* and *HMOX-1* (encoding glucose 6 phosphate dehydrogenase, NAD(P)H: quinone oxidoreductase-1 and heme oxygenase-1, respectively; Fig. 3**C**).

Taken together, exposure of cells to DEM appears to cause nuclear accumulation of FOXO1, but this does not result in elevated levels of FOXO target gene transcripts.

### 3.2 Nuclear accumulation of FOXO1-GFP is independent of FOXO1 cysteine residues

As a thiol-reactive α,β-unsaturated carbonyl compound, DEM can be anticipated to directly interact not only with glutathione and other low-molecular mass thiols, but also with protein thiols. For example, DEM modifies cysteines of the AP-1 family transcription factor Pap1 in *S. pombe*, blocking its interaction with Crm1 (exportin-1, facilitating nuclear export of proteins through the nuclear pore complex) to result in nuclear accumulation and an enhanced target gene expression [16]. FOXO transcription factors were demonstrated to interact with coregulators through their cysteines, which thereby contribute to redox regulation of FOXO activity [9], [12], [17], [18]. Human FOXO1 contains seven cysteine residues, two of which (Cys23 and Cys612) are conserved between all mammalian FOXO isoforms, and one of which (Cys23) is right next to Thr24, whose phosphorylation by the insulin-regulated kinase Akt contributes to 14-3-3 protein-dependent nuclear export of FOXO1 [19].

**Fig. 4:**
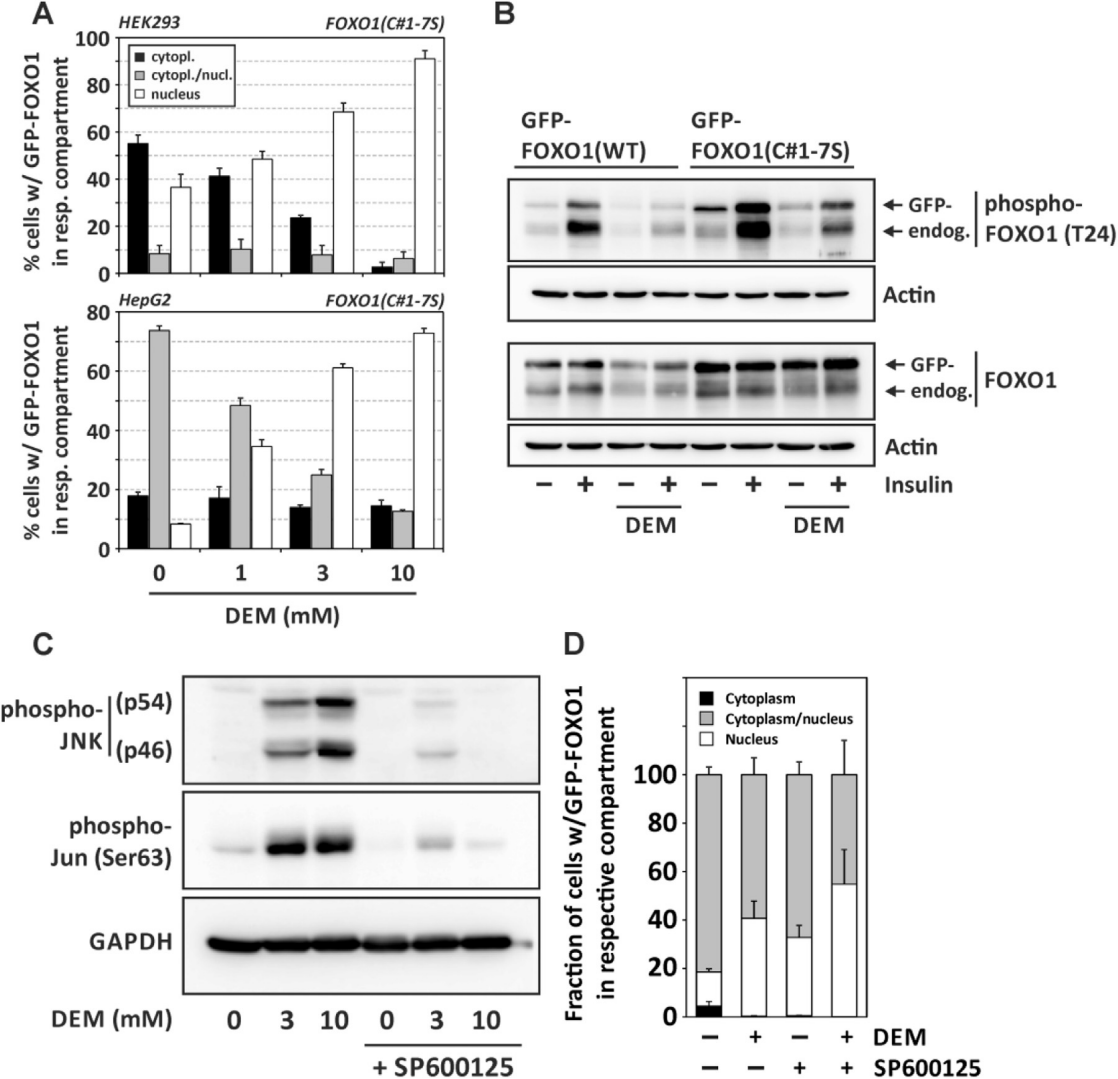
Role of FOXO1 cysteine residues and of FOXO phosphorylation in DEM response. **(A)** HEK293 or HepG2 cells transiently expressing GFP-coupled human mutant FOXO1 [FOXO1(C#1-7S)] were exposed to the given concentrations of DEM followed by analysis of subcellular distribution of GFP-FOXO1 as described in the legend to Fig. 2A. **(B)** HepG2 cells were transfected with a plasmid encoding a GFP-coupled form of FOXO1(WT) or the cysteine-deficient mutant FOXO1(C#1-7S). Cells were exposed to DEM (3 mM) or DMSO (solvent control) for 2 h, followed by 30 min exposure to insulin (100 nM) as indicated. FOXO1 and phospho-FOXO1 were detected by Western blotting, actin detection was used as gel loading control. Blots are representative of three independent experiments. **(C)** DEM stimulates JNK. HepG2 cells were incubated in the presence of 10 µM SP600125 (or DMSO as solvent control) for 1 h, followed by exposure to DEM at the given concentration for 1 h in the continued presence of SP600125 (or solvent control). Cells were lysed and subjected to SDS-PAGE and Western analysis of JNK and cJun phosphorylation. The blots shown are representative of two independent experiments. **(D)** DEM-induced nuclear accumulation of FOXO1 is independent of JNK. HepG2 cells were transfected with a plasmid expressing GFP-FOXO1 24 h prior to exposure to SP600125 (10 µM) and DEM (3 mM) as in (C). Analysis of subcellular localization of GFP-FOXO1 was done by categorizing cells according to the predominant FOXO1 distribution into “cytoplasm”, “cytoplasm/nucleus”, and “nucleus”. Subcellular distribution of GFP-FOXO1 in cells was calculated from three independent experiments. In each of these experiments, at least 198 cells were categorized per setting. Data are given as means + SD.

We generated a cysteine-deficient FOXO1 mutant with all seven cysteines mutated into serines [FOXO1(C#1-7S)] and tested for the effect of DEM on subcellular localization of this FOXO1 form. DEM causes a concentration-dependent nuclear accumulation of mutant (cysteine-deficient) EGFP-FOXO1 in both HEK293 and HepG2 cells that is indiscernible from the effects observed with wildtype FOXO1 (Fig. 4**A**).

These data suggest that DEM does not interfere with FOXO1 thiols to elicit nuclear accumulation, and they further imply that FOXO1 nuclear accumulation is independent of any transient (intra- or intermolecular) disulfide formation of FOXO1 that might have been elicited by an oxidative environment established by DEM.

### 3.3 Role of FOXO phosphorylation in DEM-induced nuclear accumulation of GFP-FOXO1

FOXO subcellular localization is extensively controlled at the level of posttranslational modification (for review, see [6]). In order to test for insulin-induced phosphorylation of mutant FOXO1 and the effect of DEM on insulin-induced FOXO1 phosphorylation, we transiently transfected GFP-coupled wildtype FOXO1 [FOXO1(WT)] or cysteine-deficient FOXO1(C#1-7S) into HepG2 cells that were then exposed to DEM and/or insulin. The use of GFP-coupled FOXO1 forms allowed us to discern endogenous (wildtype) FOXO1 from overexpressed FOXO1 by SDS-PAGE/Western blotting. Two major observations resulted from this experiment: insulin induced a strong phosphorylation of FOXO1 at Thr24 in all cases: endogenous FOXO1, overexpressed FOXO1(WT) and overexpressed mutant FOXO1. Secondly, preincubation with DEM rendered cells refractory to insulin-induced FOXO1 phosphorylation, irrespective of cysteines being present in FOXO1 or not (Fig. 4**B**). The absence of FOXO1 cysteine residues neither affects FOXO1 susceptibility to insulin-induced phosphorylation, nor does it alter the DEM-induced attenuation of insulin-induced phosphorylation. However, the observation of a DEM-induced attenuation of insulin-induced FOXO1 phosphorylation may provide a first explanation for the DEM-induced nuclear accumulation of FOXO1, as its phosphorylation by Akt is a prerequisite for insulin-induced nuclear exclusion of FOXO1.

In order to test whether exposure to DEM merely causes a general inhibition of phosphorylation cascades, we investigated stimulation of the stress-activated cJun-N-terminal kinases (JNK) by DEM. Indeed, JNK were strongly stimulated in HepG2 cells exposed to DEM, as demonstrated by dual phosphorylation (indicative of their activation) of JNK 46 kDa and 54 kDa isoforms. Likewise, their immediate substrate, cJun, was phosphorylated under these conditions (Fig. 4**C**).

Whereas FOXO phosphorylation by protein kinase Akt results in FOXO nuclear exclusion, cJun-N-terminal kinases were previously shown to support nuclear accumulation of FOXO4 upon exposure to hydrogen peroxide [20]. Therefore, we tested for a role of JNK in the DEM-induced nuclear accumulation observed here. As no phosphorylation of FOXO1 by JNK has been observed yet, we expected that JNK are not involved in FOXO1 nuclear accumulation. This is, indeed, what our data suggest: Both JNK and cJun phosphorylation was inhibited in the presence of SP600125, an inhibitor of stress-activated protein kinases, such as JNK and p38 [21]. In contrast, DEM-induced FOXO1 nuclear accumulation was not attenuated in the presence of SP600125. Instead, basal nuclear accumulation of GFP-FOXO1 appears to be slightly stimulated by this inhibitor (Fig. 4**D**, white bar sections). In summary, DEM, although stimulating stress-induced signaling to result in activation of JNK, attenuates FOXO phosphorylation induced by insulin. While the former is not involved in FOXO1 nuclear accumulation, the latter is in line with the lack of nuclear exclusion of FOXO1 in cells exposed to DEM.

### 3.4 DEM interferes with nuclear export of FOXO1

**Fig. 5:**
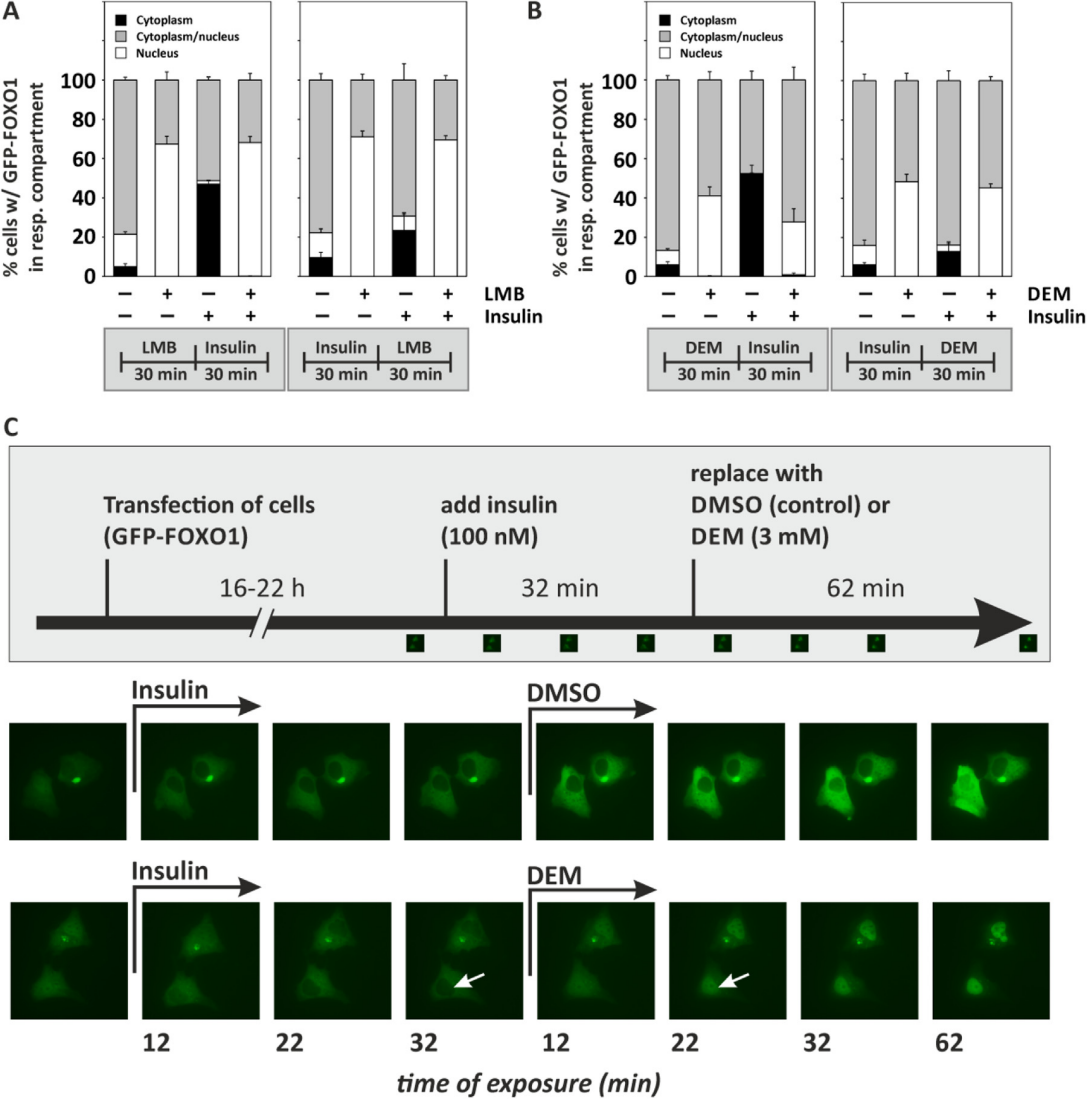
Analysis of subcellular localization of FOXO1 upon exposure to Leptomycin B or DEM. HepG2 cells were transfected with an expression plasmid coding for GFP-FOXO1, followed by exposure to the given compounds and analysis of subcellular distribution of GFP-FOXO1 as described before. **(A)** Cells were exposed to Leptomycin B (LMB) at 30 ng/mL for 30 min, followed by addition of insulin (100 nM) for another 30 min. In the panel on the right, insulin was added first (30 min), followed by LMB (30 min) prior to analysis of GFP-FOXO1 subcellular localization. **(B)** Cells were treated as in (A), with DEM (10 mM) instead of LMB. Subcellular distribution of EGFP-FOXO1 in cells was calculated from three independent experiments. On average, 210 (min: 124, max: 267) cells were categorized per condition in each of the independent experiments. Data are given as means + SD. **(C)** HepG2 cells expressing EGFP-FOXO1 were exposed to insulin (causing nuclear exclusion) and DEM (3 mM) or solvent control (DMSO) for the indicated times. Analysis of subcellular distribution of EGFP-FOXO1 was done as described in Materials and Methods.

It was previously reported that DEM blocks nuclear export through interaction with exportin Crm (chromosome region maintenance)-1 and nucleoporins [22]. In order to test whether this may apply also to FOXO1, we blocked Crm-1-dependent nuclear export in cells overexpressing GFP-FOXO1 using leptomycin B (LMB) and analyzed the resulting subcellular distribution of GFP-FOXO1. As seen in Fig. 5**A**, nuclear accumulation of GFP-FOXO1 was elicited (white bar sections), even within 30 min of exposure. In contrast, insulin elicited extensive nuclear exclusion (Fig. 5A, increase in cytoplasmic localization, black bar sections). The latter was prevented by LMB, confirming that insulin-induced nuclear exclusion of GFP-FOXO1 requires Crm-1. LMB not only prevented nuclear exclusion of GFP-FOXO1, but it also reversed nuclear exclusion already elicited by an insulin exposure prior to addition of LMB (Fig. 5A, right panel), indicating that an equilibrium between nuclear and cytoplasmic GFP-FOXO1 exists that was shifted to the nuclear side.

DEM behaved like LMB (Fig. 5**B**): it elicited the same quick response in favor of GFP-FOXO1 nuclear accumulation within 30 min, and it both prevented (left panel) and reversed (right panel) insulin-induced nuclear exclusion. The latter is illustrated using selected cells in Fig. 5**C**: exposure of cells to insulin caused visible nuclear exclusion of GFP-FOXO1 within 12 min. Exchange of insulin for DEM (but not the solvent control, DMSO) caused a reversal, and GFP-FOXO1 was forced into the nuclei (see, for example, white arrows prior to, and after, addition of DEM).

**Fig. 6:**
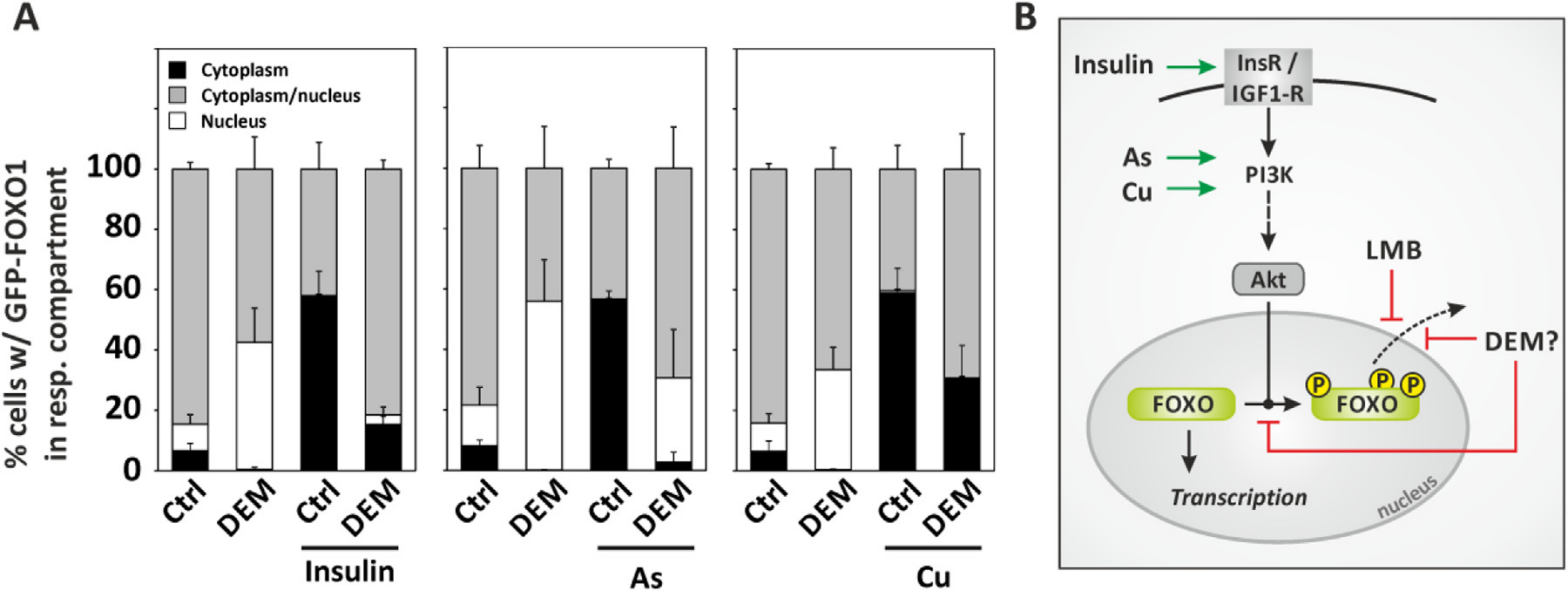
Analysis of subcellular localization of FOXO1 upon exposure to stressful stimuli. HepG2 cells were transfected with an expression plasmid coding for GFP-FOXO1, followed by exposure to the given compounds and analysis of subcellular distribution of GFP-FOXO1 as described before. **(A)** Cells were treated with DEM (2 mM) for 30 min, followed by addition of insulin (left), sodium arsenite (100 µM, middle) or copper sulfate (10 µM, right) for another 60 min. Data are means of at least three independent experiments + SD. On average, 149 (min: 92, max: 240) cells were categorized per condition in each of the independent experiments. **(B)** Schematic summary of findings: DEM causes nuclear accumulation of FOXO1 by blocking its nuclear exclusion. Nuclear exclusion elicited by stimuli such as insulin, copper ions or arsenite is attenuated by DEM. Abbreviations in scheme: InsR, insulin receptor; IGF1-R, insulin-like growth factor.

Should DEM inhibit nuclear export, this would not only interfere with insulin-induced effects on subcellular localization of FOXO1 as seen in Fig. 5C, but it would similarly affect insulin-like effects elicited by other stimuli. For example, we have previously demonstrated that arsenite [10], [23] and copper ions [11], [24] elicit insulin-like effects on FOXO transcription factors, with FOXO1 nuclear exclusion being one consequence of exposure. We therefore preincubated HepG2 cells with DEM for 30 min, followed by a 60 min-exposure to insulin or insulin mimetic stimuli, arsenite or Cu^2+^. DEM was used at a lower concentration of 2 mM in order to prevent the additional stress imposed by a subsequent exposure to copper ions from impairing cellular integrity. The lower concentration of DEM also elicited a nuclear accumulation of GFP-FOXO1, and it attenuated insulin-induced nuclear exclusion (Fig. 6**A**, left bar graph panel), albeit not as effectively as the higher concentrations employed in Fig. 5B. DEM attenuated arsenite-induced nuclear exclusion (even more effectively than that elicited by insulin; Fig. 6**A**, middle bar graph panel) and also attenuated the effect of copper ions, with the fraction of cells carrying GFP-FOXO1 predominantly in their cytoplasmic compartment lower than without DEM (Fig. 6**A,** right panel).

Together, these data suggest that DEM interferes with nuclear exclusion stimulated by agents acting on FOXO1 in an insulin-like fashion (Fig. 6**B**).

## 4 Discussion

Exposure of cells to xenobiotics comes with an activation of xenosensors, such as nuclear receptor xenosensors (e.g., the constitutive androstane receptor or pregnane X-receptor), the aryl hydrocarbon receptor, or Nrf2. FOXO transcription factors were previously demonstrated to interact with many of these, establishing a link between environmental stimuli and metabolic regulation in mammalian cells (for review, see [25]).

Here, we investigated the effects of a known Nrf2 activator, diethyl maleate (DEM), on FOXO transcription factors in mammalian cells: We demonstrate that exposure of mammalian cells to DEM causes nuclear accumulation of FOXO1 transcription factor, but no stimulation of FOXO-dependent gene expression. We hypothesize that FOXO1 subcellular distribution is affected through interference of DEM with FOXO1 phosphorylation (Fig. 4) and with the nuclear export machinery (Fig. 5, Fig. 6), as previously suggested by data in HeLa cells [22]. In line with this hypothesis, (i) accumulation of FOXO1 was observed in cells exposed to the nuclear export inhibitor targeting Crm1, leptomycin B (Fig. 5A), in a fashion similar to DEM (although at much lower concentrations than with DEM). (ii) Moreover, DEM both blocks and reverses insulin-induced nuclear exclusion of FOXO1 (Fig. 5B, C) and (iii) nuclear exclusion elicited by stimuli such as arsenite and copper ions (Fig. 6A).

Interestingly, DEM was also shown to attenuate classical (NLS-dependent) nuclear import in HeLa cells, with contributions of phosphoinositide 3-kinase and MAPK/ERK kinase (MEK) that target proteins involved in nuclear transport [26]. We did not observe this effect in HepG2 cells: a clear FOXO1 nuclear accumulation predominated whenever cells were exposed to DEM. Moreover, known Nrf2 target genes were upregulated (Fig. 3), implying that Nrf2 nuclear translocation was not impaired but rather enabled by exposure to DEM.

DEM was described previously as a Nrf2 activator, interacting with Cys151 of Keap1, thereby interfering with Keap1-mediated Nrf2 degradation [27], [28]. In line with these data, we here find the upregulation of Nrf2 target gene mRNAs (Fig. 3). FOXO1 contains seven cysteine residues, at least one of which is involved in FOXO1 activity as a transcription factor [12]. An interaction of DEM with FOXO1 cysteines analogous to DEM interaction with Keap1 would therefore appear a possible explanation for the effect of DEM on FOXO signaling. However, neither FOXO1 nuclear transport in response to DEM nor FOXO1 phosphorylation in response to insulin appear to be affected by the absence of FOXO1 cysteines, as demonstrated in experiments using the overexpression of a FOXO1(C#1-7S) mutant (Fig. 4). Nevertheless, it may be speculated that nuclear FOXO1, which does not appear to be activated beyond control levels under the influence of DEM, but rather appears to lose activity under these conditions (Fig. 3A, B), is a target of DEM. This hypothesis would be in line with the observed loss of transactivation activity of cysteine-deficient FOXO1 [12].

Both FOXO and Nrf2 signaling are triggered by stressful stimuli. An interaction between Nrf2 and FOXO transcription factors (be it functional or even physical) would therefore make sense, and such interaction was indeed described in the literature (for review, see [25]); for example, it was demonstrated in human tumor cell lines cells that FOXO3 stimulates the transcription of the Keap-1 gene [29], regulating Keap-1 protein levels; FOXO3 would thus attenuate Nrf2 action by elevating Keap-1 levels, whereas FOXO inactivation (e.g. through insulin-induced activation of Akt) would tend to stimulate Nrf2. The observed DEM-induced regulation of FOXO1 and Nrf2 in opposite directions (Fig. 3) would therefore be in line with published observations on the relationship between FOXO and Nrf2 signaling.

The situation was different in *C. elegans*: whereas DEM caused nuclear accumulation of DAF-16 (the FOXO ortholog in *C. elegans*; see Fig. 2D), apparently imitating the effect observed in cultured human cells, the exposure of worms to low DEM concentrations resulted in an enhanced stress resistance and elevated lifespan. Moreover, extension of lifespan requires both DAF-16 and SKN-1 (the *C. elegans* ortholog of Nrf2) [2]. Although the nature of DAF-16/SKN-1 cooperation remains to be explored, it is obvious that the effect of DEM that was observed in HepG2 or HEK293 cells is different from the *in vivo* model in that no clear FOXO activation was detected.

In summary, DEM triggers FOXO1 nuclear accumulation but not activation, whereas Nrf2 activity is stimulated. Nuclear accumulation appears to be stimulated by interference with FOXO1 nuclear export and is independent of an interaction of DEM with FOXO1 cysteine residues.
